# Degenerative Spinal Stenosis and Ipsi-Contralateral Decompression: Presentation of a Surgical Technique and Clinical Cases

**DOI:** 10.7759/cureus.65737

**Published:** 2024-07-30

**Authors:** Asen Cekov, Marin Guentchev, Vladimir Nakov, Anastas Kanev, Ivan Tarev

**Affiliations:** 1 Department of Neurosurgery, Acibadem City Clinic Tokuda Hospital, Sofia, BGR

**Keywords:** minimally invasive spine surgery, surgical management, ipsi-contralateral decompression, lumbar spinal stenosis (lss), degenerative lumbar spine

## Abstract

Lumbar spinal stenosis is a widespread condition that significantly affects the quality of life in elderly individuals. Conservative therapy has a positive effect on patients whose primary symptom is pain. However, in severe cases with the presence of hypesthesia and paresis, surgical treatment comes into consideration. The aim of surgery is to decompress the neurovascular elements compressed by the narrowed spinal canal while preserving spinal stability. Conventional laminectomy, with or without fusion, has been considered effective for the treatment of this pathology, but its drawbacks are significant, including tissue trauma, secondary instability, and a substantial percentage of reoperations due to complications. In recent years, various minimally invasive spine surgery techniques have emerged, showing comparable results to laminectomy decompression in terms of relieving symptomatic spinal stenosis. Additionally, these techniques offer significant benefits such as minimal tissue trauma, reduced complication rates, and shorter operative time and recovery periods. Given the continuous development and improvement, minimally invasive surgery is expected to widely replace traditional open surgery for the treatment of lumbar stenosis in the future.

In this article, we present our experience in the surgical treatment of patients with degenerative lumbar stenosis, detailing the technique of the minimally invasive procedure we utilize and highlighting some of the clinical cases in which it has been applied.

## Introduction

Lumbar spinal stenosis is a common pathology in elderly individuals, resulting from degenerative aging processes affecting the bony and ligamentous structures of the spine [1]. In the USA, it is the most frequent cause of spinal surgery in patients over 65 years of age [2]. Epstein reported that the prevalence of absolute lumbar stenosis is 47.2% among patients aged 60-69 years, and this percentage increases with age [3]. The characteristic clinical syndrome includes low back pain, pain and numbness in the lower limbs, and leg weakness, with more severe clinical manifestations leading to a significant decrease in the quality of life of patients. Since sudden changes in symptomatology are uncommon [4], non-surgical treatment is usually chosen initially for people with mild or moderately expressed symptoms. Surgical decompression offers an advantage over non-surgical treatment for selected patients with severe symptoms who do not respond to conservative therapy [5,6]. In recent years, minimally invasive decompression methods have increasingly been used in practice, showing similar efficacy to conventional laminectomy in alleviating symptoms, and significantly reducing operative time, hospital stay, and the risk of inducing spinal instability. At our clinic, we use a minimally invasive ipsilateral-contralateral decompression surgical method, detailed in the discussion section.

## Case presentation

Case 1

**Figure 1:**
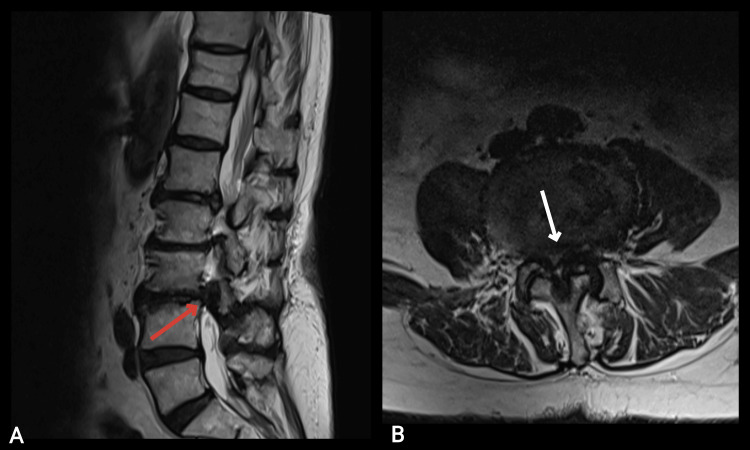
Preoperative MRI of an 85-year-old woman showing high-grade spinal stenosis in the sagittal plane (red arrow) and axial plane (white arrow) at the L3-L4 level. A: T2 sagittal; B: T2 axial.

An 85-year-old woman was admitted to the clinic with symptoms of long-standing severe pain and paresthesia, initially along the anterolateral surfaces of both legs, currently without a pronounced dermatomal pattern, which in recent months has become unbearable and unresponsive to medication. She reported weakness in both legs, especially pronounced in the last few weeks, and an inability to walk more than 15 meters independently. Imaging studies, including MRI of the lumbar spine, showed multisegmental spinal stenosis, particularly severe at the L3-L4 level, grade D according to the Schizas scale, with foraminal stenosis and radicular compression at the same level (Figure 1).

Given the patient's age, it was decided to perform a minimally invasive extended laminotomy and medial facetectomy at the L3-L4 level on the right side, followed by flavectomy and ipsi-contralateral decompression under optical magnification at the specified level on the left side. On the first postoperative day, the patient reported a noticeable reduction in pain and was discharged on the third postoperative day with significant symptom improvement.

**Figure 2:**
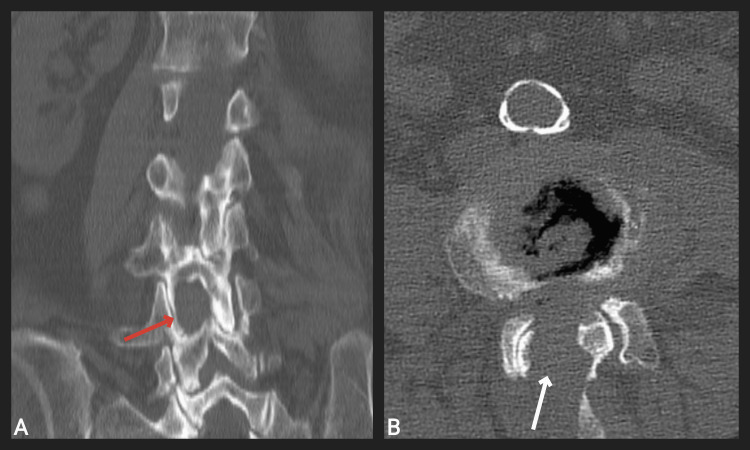
Postoperative CT scan of an 85-year-old woman showing the performed decompression in the coronal plane (red arrow) and axial plane (white arrow) at the L3-L4 level. A: Coronal plane; B: axial plane.

**Figure 3:**
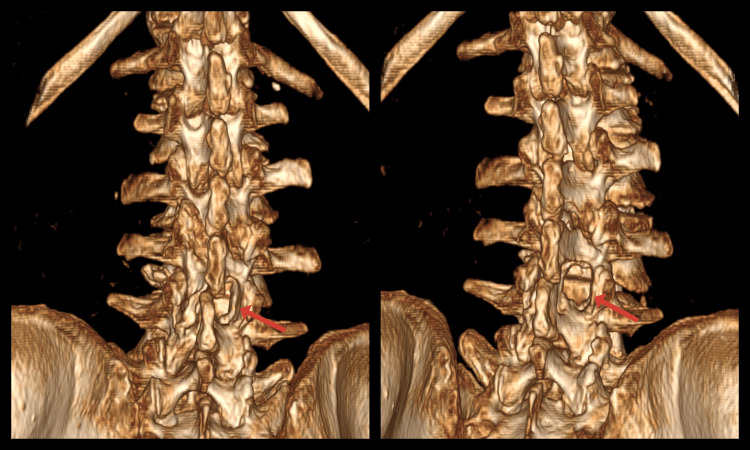
Postoperative 3D reconstructions of an 85-year-old woman showing the performed decompression (red arrows) at the L3-L4 level.

Postoperative CT of the lumbar segment of the spine with bone and 3D reconstructions demonstrated successfully performed bilateral decompression of the spinal canal via unilateral approach at the L3-L4 level (Figures 2, 3).

Case 2

An 81-year-old man was admitted to the clinic with symptoms of long-standing low back pain radiating to the anterior and posterolateral surfaces of both legs, more pronounced on the right side. Over the last two months, the pain has significantly intensified and has been difficult to manage with medication, accompanied by weakness in the lower limbs. Neurological examination showed a moderately pronounced spasm of the lumbar paravertebral muscles, and impaired plantar and dorsal flexion of the feet, more pronounced in the right lower limb. He had a positive Wasserman sign bilaterally and a positive Lasègue sign at 45 degrees on the right and 70 degrees on the left.

**Figure 4:**
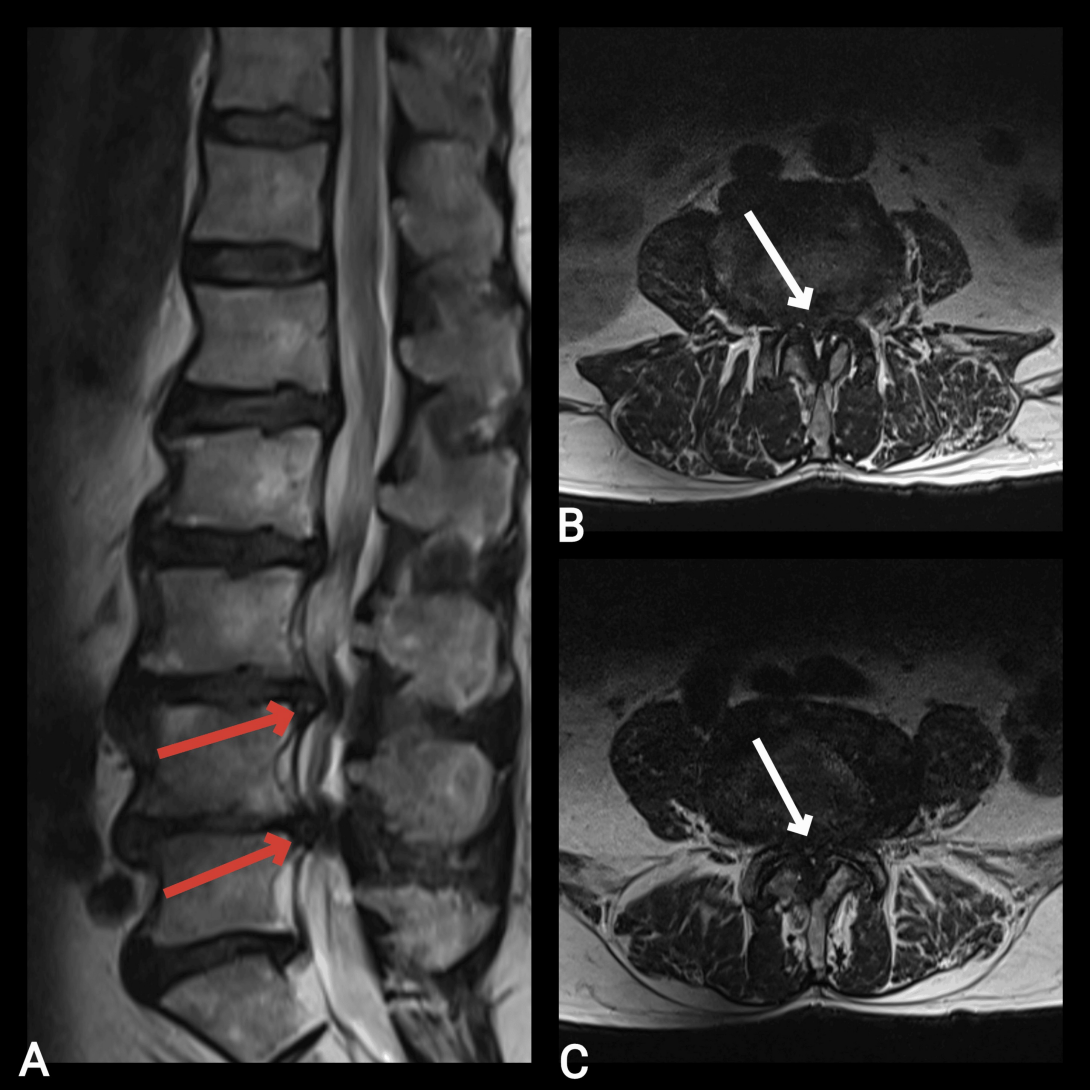
Preoperative MRI of an 81-year-old male showing high-grade spinal stenosis in the sagittal plane (red arrow) and axial plane (white arrow) at the L3-L4 and L4-L5 levels. A: T2 sagittal; B: T2 axial at the L3-L4 level; C: T2 axial at the L4-L5 level.

MRI of the lumbar spine showed high-grade spinal stenosis at the L3-L4 level, grade C according to the Schizas scale, and at the L4-L5 level, grade D according to the Schizas scale (Figure 4).

Given the lack of response to conservative treatment and the worsening neurological symptoms, the patient underwent surgical treatment - minimally invasive extended laminotomy with medial facetectomy at the L3-L4 and L4-L5 levels on the right side. Under optical magnification, flavectomy was performed, followed by ipsilateral-contralateral decompression at the described levels on the left side. On the first postoperative day, the patient reported a significant reduction in pain symptoms and was discharged from the clinic on the fourth postoperative day.

**Figure 5:**
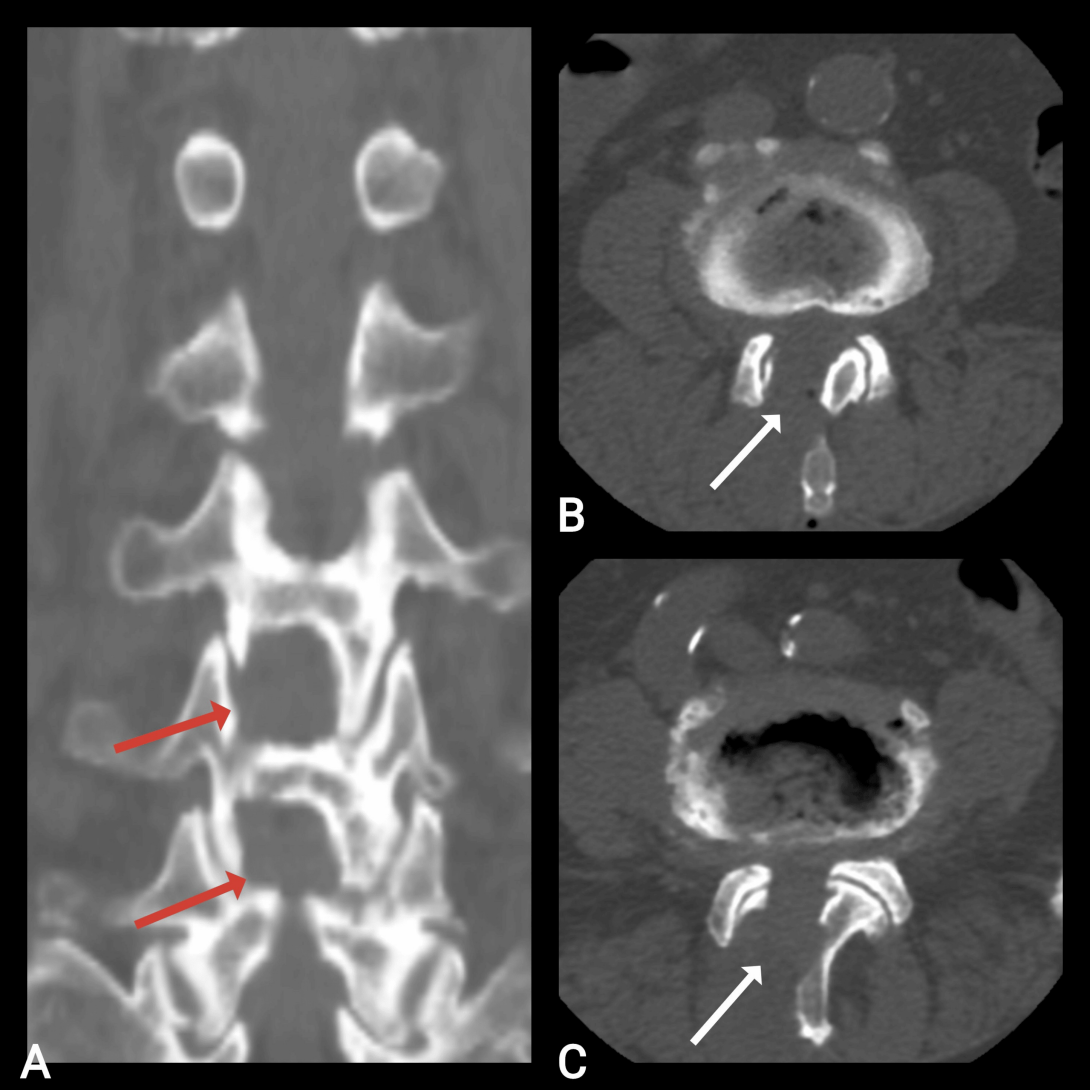
Postoperative CT scan of an 81-year-old male showing the performed decompression in the coronal plane (red arrow) and axial plane (white arrow) at the L3-L4 and L4-L5 levels. A: Coronal plane; B: axial plane at the L3-L4 level; C: axial plane at the L4-L5 level.

**Figure 6:**
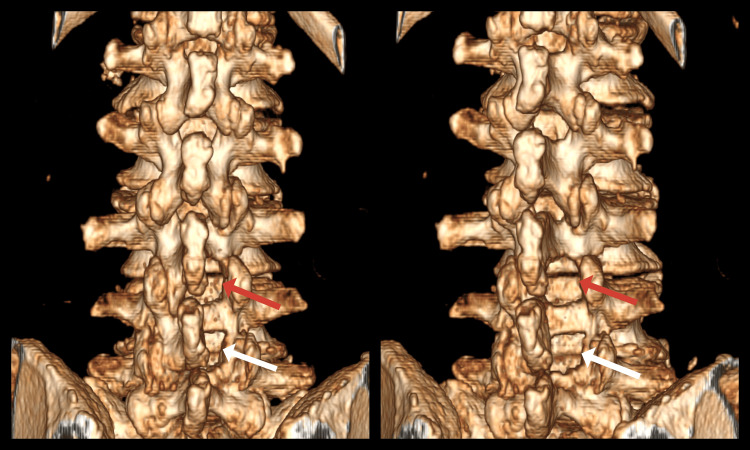
Postoperative 3D reconstruction of an 81-year-old male showing the performed decompression (red arrows) at the L3-L4 and L4-L5 levels.

Postoperative CT of the lumbar spinal canal with bone and 3D reconstructions demonstrated successfully performed minimally invasive bilateral decompression at the L3-L4 and L4-L5 levels (Figures 5, 6).

## Discussion

Lumbar spinal stenosis is a pathological condition causing the narrowing of the spinal canal, which compresses the nerve and vascular structures passing through it. It is usually a degenerative condition affecting elderly individuals, though its occurrence is not excluded in younger people. The narrowing of the spinal canal generally occurs slowly over many years or decades. The intervertebral discs become denser over the years, leading to a loss of their height and potentially causing the protrusion of the hardened disc into the spinal canal. The facet joints undergo degenerative changes leading to bone hypertrophy, which also contributes to the reduction of the canal's overall size [7,8]. The hypertrophy of the ligamentum flavum also plays an important role in the pathogenesis of spinal stenosis [9]. The appearance of bone spurs is also possible. All these factors can contribute to the narrowing of the central canal, which can cause neurological symptoms such as radicular pain, impaired sensation in the lower extremities, neurogenic claudication, and varying degrees of motor function disability in the legs, as well as loss of normal bowel or bladder function. A significant portion of patients with lumbar stenosis are asymptomatic, but those with at least a 50% reduction in canal width often exhibit symptoms of neurogenic claudication [10]. It is important to distinguish neurogenic claudication from intermittent claudication, which is due to impaired blood supply to the legs and insufficient blood flow to the lower extremities muscles due to peripheral arterial insufficiency.

A combination of medication, posture management, and various exercises can be beneficial for many patients with spinal stenosis and mild symptoms. When these measures do not help, surgical methods come into consideration. Approximately 10%-15% of patients require surgery [11]. In most cases, the problem can be resolved by performing decompression alone, with stabilization of the respective segment reserved for cases with radiographic and clinical evidence of instability.

The posterior paraspinal muscles mainly consist of the multifidus, longissimus, and iliocostalis muscles. The multifidus muscle is important for the stability of the lumbar region, with its fascicles attaching to the spinous processes of the vertebrae through so-called insertions, and innervated by the medial branches of the dorsal rami of the spinal nerves. In a classical laminectomy, these insertions must be detached from the spinous processes bilaterally, and the multifidus muscle subjected to retraction to visualize the lamina of the vertebra. Numerous studies show that postoperative chronic back pain and multifidus muscle atrophy are due to prolonged muscle retraction, disrupting arterial blood supply, and damage to the posterior branches of the dorsal rami [12,13]. Periodic release of the paraspinal muscle retractors during surgery is recommended to improve muscle perfusion [14]. To reduce postoperative back pain caused by paraspinal muscle atrophy, various modified minimally invasive procedures have been described [15-18].

Our experience shows that hypertrophy of the ligamentum flavum is one of the leading causes of symptomatic spinal canal stenosis, and its bilateral removal leads to good decompression of the spinal cord and dural sac, which is sufficient to alleviate symptoms in most cases. Therefore, the technique of unilateral laminotomy followed by bilateral decompression (ipsi-contralateral decompression) is our preferred surgical method. The main objective of this technique is to accomplish effective decompression while minimizing the removal of bony structures, thereby maintaining optimal spinal stability.

After induction of general anesthesia, the patient is placed in a prone position on a padded frame to open the interlaminar space and reduce intra-abdominal pressure. The level of decompression is determined by lateral fluoroscopy and marked with a needle. We usually use a midline or 1 cm lateral to the midline incision, accessing from the more symptomatic side. The following steps involve dissection of the thoracolumbar fascia and its incision, separation, and detachment of the paravertebral muscle insertions from the spinous process and lamina of the vertebra, and placement of a retractor. The retractor's position is verified by lateral fluoroscopy.

The next stage of surgery is performed under microscopic magnification. After identifying the facet joint, we begin laminotomy and medial facetectomy using a high-speed drill. Care is taken to avoid excessive bone resection of the facet. Upon completion of ipsilateral bone decompression, the ipsilateral ligamentum flavum is removed using a Kerrison rongeur. At this stage, the underlying dura mater and ipsilateral nerve root are visualized. Foraminal decompression can be performed with the Kerrison rongeur.

The operating table is tilted in the direction opposite to the surgical access, or the microscope is turned medially for better visualization of the contralateral part of the spinal canal. The procedure continues with trimming the lower part of the spinous process and visualizing the ventral part of the contralateral lamina. It is particularly important to preserve the contralateral ligamentum flavum, which protects the dura until complete bony decompression is achieved. Decompression of the contralateral recess can be accomplished by trimming the medial part of the facet joint with a high-speed drill or Kerrison if necessary. Visualization of the contralateral nerve root is ensured by resecting the remaining ligamentum flavum. In most cases, removing the contralateral ligamentum flavum while preserving the contralateral facet joint is sufficient to ensure good decompression and symptom relief. This is followed by thorough hemostasis and revision of the surgical field, removal of the retractor, and layered closure of the surgical access.

Recently, minimally invasive decompression techniques have become increasingly popular among spinal surgeons, with studies showing comparable results to standard laminectomy [8,19]. Rahman et al. [18] report a reduction in the duration of minimally invasive procedures compared to conventional laminectomy by 37 to 47 minutes. Preserving at least half of both facet joints is crucial to avoid iatrogenic instability [20].

## Conclusions

Spinal canal stenosis is a degenerative condition most commonly observed in elderly individuals. When conservative approaches prove unsuccessful, surgical treatment methods become a consideration. Minimally invasive bilateral decompression is comparable to conventional laminectomy in alleviating patient symptoms and offers additional advantages, such as reduced operative time and hospital stay, lower trauma, and decreased complication rates. Our experience with the described technique indicates that the need for stabilization following its correct application is minimized.
